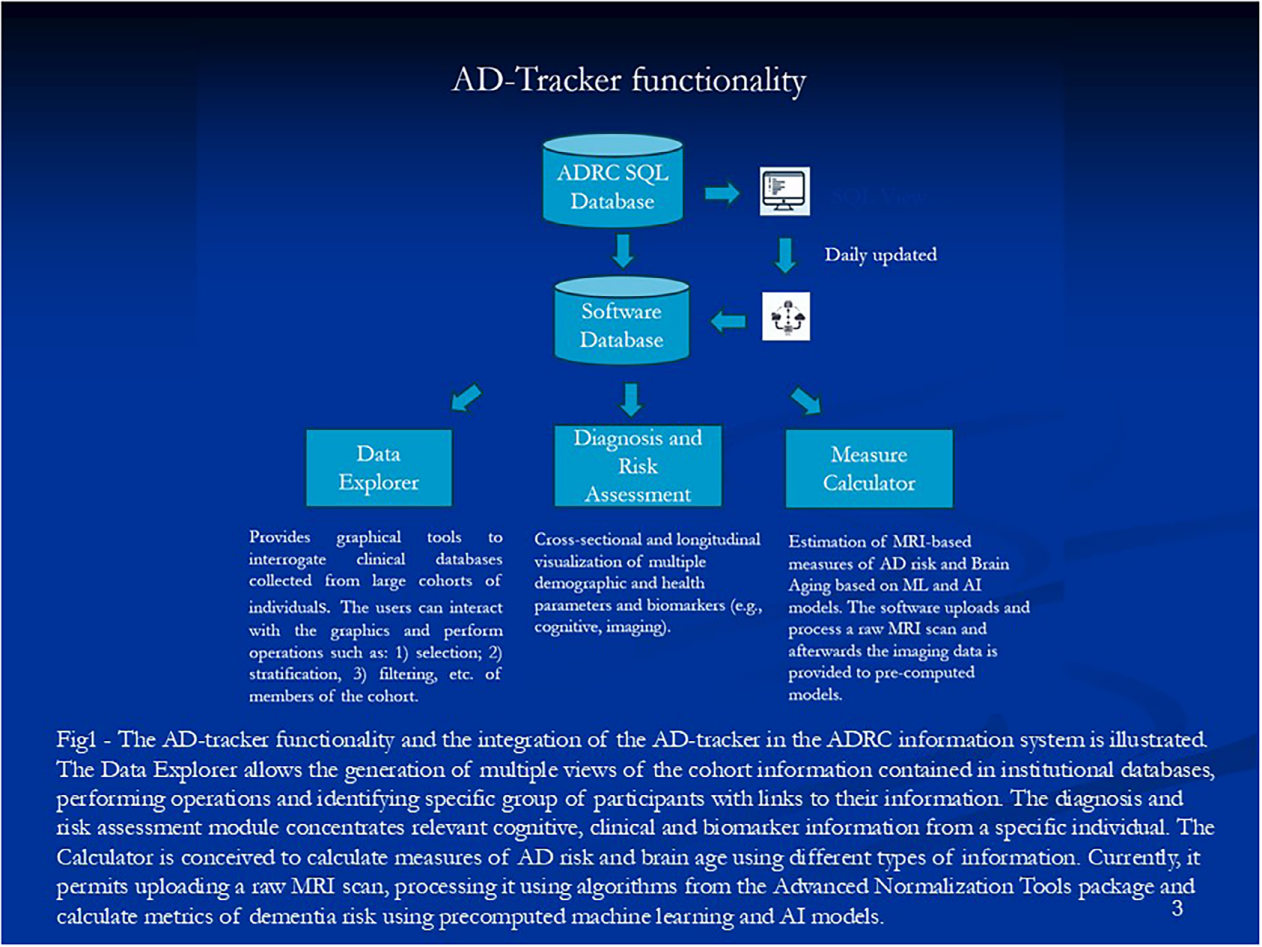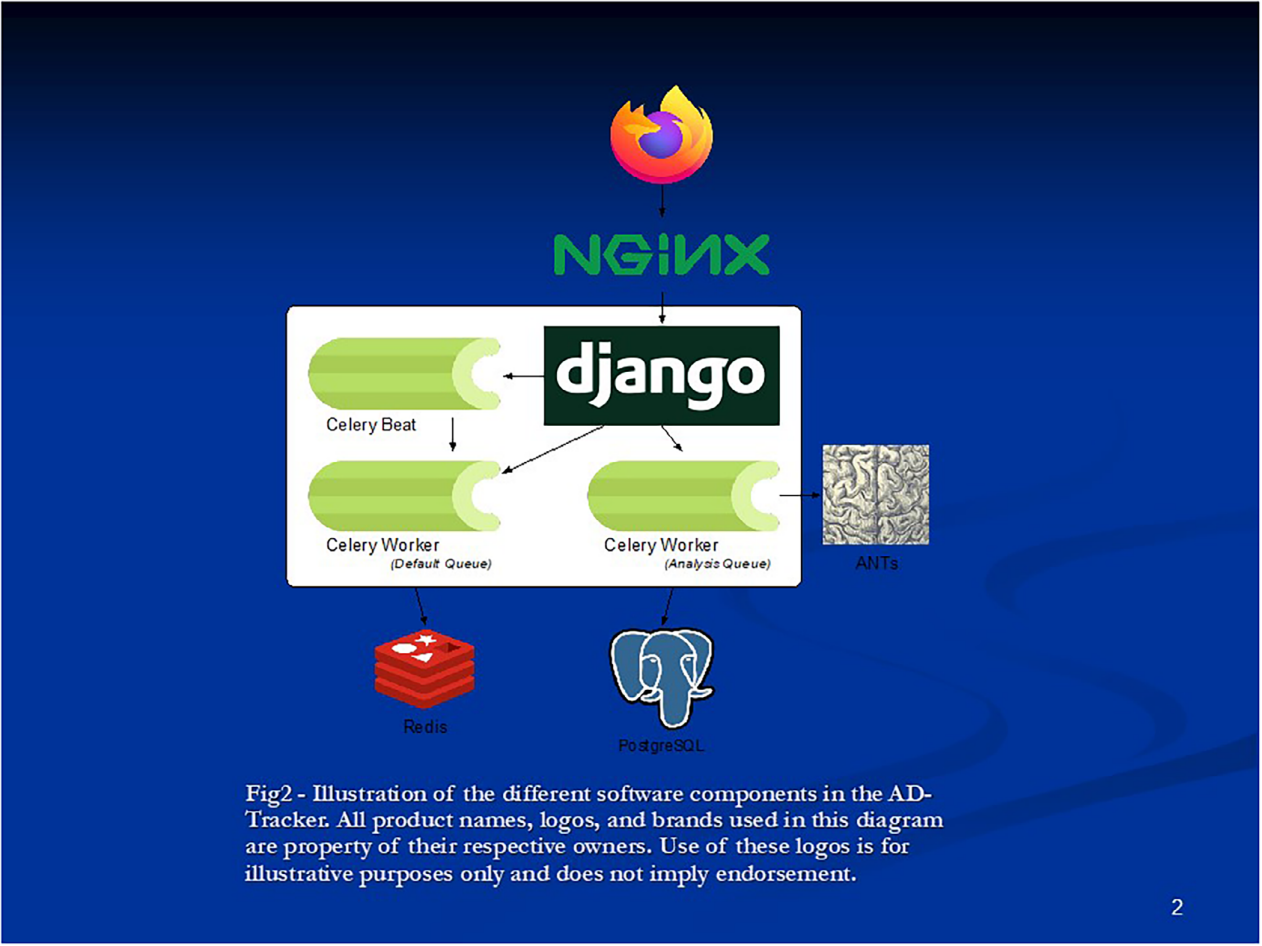# AD‐tracker: a software application to support clinical decision‐making and research ‐ Design and general functionality

**DOI:** 10.1002/alz70860_100480

**Published:** 2025-12-23

**Authors:** Ryan Barnard, Sarah A. Gaussoin, John P Hepler, Syam Gadde, Melissa M. Rundle, Marc D. Rudolph, Lauren W Green, Courtney L. Sutphen, Ganesh M. Babulal, Michael W Lutz, Suzanne Craft, Mark A. Espeland, Trey R. Bateman, Ramon Casanova

**Affiliations:** ^1^ Wake Forest University School of Medicine, Winston‐Salem, NC, USA; ^2^ Wake Forest University School of Medicine, Winston Salem, NC, USA; ^3^ Duke University, Durham, NC, USA; ^4^ Wake Forest Alzheimer's Disease Research Center, Winston‐Salem, NC, USA; ^5^ Wake Forest School of Medicine, Winston‐Salem, NC, USA; ^6^ Washington University School of Medicine, Saint Louis, MO, USA; ^7^ Duke Department of Neurology, Durham, NC, USA; ^8^ Duke University School of Medicine, Durham, NC, USA; ^9^ Wake Forest University, Winston‐Salem, NC, USA

## Abstract

**Background:**

Clinical and research experts often struggle to access and fully utilize the large amount of health parameters and biomarkers available in research and clinical databases. Moreover, the data analyzed by researchers frequently represent static snapshots in time, quickly becoming outdated. In addition, sophisticated analytics to deal with high‐dimensional data based on Machine Learning (ML) and Artificial Intelligence (AI) are performed off‐line by specialized personnel, which limits their wider use. We have developed the AD‐tracker software to address these challenges. The first version is a modular and containerized application designed to be utilized across Alzheimer's Disease Research Centers.

**Methods:**

AD‐tracker integrates with institutional databases, enabling up‐to‐date access to longitudinal clinical, cognitive, and biomarker data. It presents a graphical user interface for intuitive database exploration and patient‐specific risk assessment across three modules (Figure 1): (1) The Risk Assessment Module visualizes individual patient data, including demographics, cognitive scores, biomarkers, and longitudinal trajectories alongside comparisons to cognitively normal cohorts. (2) The Data Explorer Module provides customizable scatterplots that allow direct graphical interaction for patient subset selection and stratification, or filtering based on user‐defined criteria. (3) The Risk Calculator Module employs the Advanced Normalization Tools package and ML and AI models to derive measures of neuroanatomic risk and brain age from structural MRI scans. AD‐tracker is built on a scalable architecture comprising a Django backend, PostgreSQL database, Redis for task brokering, and Celery for parallelized processing of resource‐intensive tasks Figure 2). *The application supports Linux‐based deployments using Docker or Podman*.

**Results:**

A version of the application is currently undergoing testing and refinement at Wake Forest University School of Medicine Alzheimer's Disease Center, and the portability of the software has been demonstrated by successful installation at external sites. Preliminary evaluations of the tool's utility have demonstrated its potential to facilitate rapid access to clinical, cognitive, and biomarker data for clinicians and researchers.

**Conclusions:**

The AD‐tracker has been designed to combine the power of visualization and complex computations while providing close to real time access to data to a wide range of users. The AD‐tracker is an AI project being developed for the research community at large.